# New body mass index for predicting prognosis in patients with antineutrophil cytoplasmic antibody‐associated vasculitis

**DOI:** 10.1002/jcla.24357

**Published:** 2022-03-21

**Authors:** Jung Y. Pyo, Sung S. Ahn, Lucy E. Lee, Jason J. Song, Yong‐Beom Park, Sang‐Won Lee

**Affiliations:** ^1^ 37991 Division of Rheumatology Department of Internal Medicine Yonsei University College of Medicine Seoul Korea; ^2^ 37991 Institute for Immunology and Immunological Diseases Yonsei University College of Medicine Seoul Korea

**Keywords:** antineutrophil cytoplasmic antibody‐associated vasculitis, conventional BMI, new BMI, poor outcomes, underweight

## Abstract

**Objectives:**

Body mass index (BMI) is a known indicator of all‐cause mortality. However, conventional BMI does not reflect the three‐dimensional human body. To overcome this limitation, a new BMI has been proposed that provides a closer approximation of real human body shape. This study investigated the associations between the new BMI and poor outcomes in patients with antineutrophil cytoplasmic antibody‐associated vasculitis (AAV).

**Method:**

We retrospectively reviewed the medical records of 242 patients with AAV in a single tertiary medical center. Based on the new BMI, the patients were categorized into four groups: underweight (<18.5 kg/m^2.5^), healthy weight (18.5 to <25.0 kg/m^2.5^), overweight (25.0 to <30.0 kg/m^2.5^), and obese (≥30.0 kg/m^2.5^). The association among the new BMI and death, relapse, end‐stage renal disease (ESRD) development, cerebrovascular accident, and cardiovascular disease was analyzed.

**Results:**

The underweight group, according to the new BMI, had higher hazard ratios (HRs) for all‐cause mortality (HR: 3.180, 95% confidence interval [CI]: 1.134–8.922, *p* = 0.028), relapse (HR: 2.141, 95% CI: 1.019–4.368, *p* = 0.036), and ESRD development (HR: 2.729, 95% CI: 1.190–6.259, *p* = 0.018) than the healthy weight group. However, according to the conventional BMI, there were no differences in the risks for all poor outcomes between the underweight and healthy weight groups. Multivariate logistic regression analysis demonstrated that being underweight, according to the new BMI, was an independent risk factor for all‐cause mortality (HR: 5.285; 95% CI: 1.468–19.018; *p* = 0.011).

**Conclusion:**

Being underweight, according to the new BMI, is associated with poor outcomes in patients with AAV.

## Graphical abstract

The new BMI provides a closer approximation of the real human body shape. Underweight by the new BMI is associated with poor outcomes in AAV, whereas underweight by the conventional BMI is not. Evaluating the nutritional status of patients with AAV during diagnosis using the new BMI may be useful as a prognostic factor.

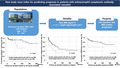

## 1 INTRODUCTION

Antineutrophil cytoplasmic antibody (ANCA)‐associated vasculitis (AAV) is a fatal disease that includes microscopic polyangiitis (MPA), granulomatosis with polyangiitis (GPA), and eosinophilic granulomatosis with polyangiitis (EGPA).^1^, ^2^ The causes of death within the first year include both vasculitis and infection, whereas those after the first year include cardiovascular disease (CVD), malignancy, and infection.^3^, ^4^ Prognosis has dramatically improved since the introduction of corticosteroids and cyclophosphamide, with 1‐year mortality rate of 80% in untreated patients to a 5‐year survival rate of 75% in treated patients.^3^, ^5^ However, the mortality rate remains as high as 10%–15%, despite sufficient treatment with newly suggested therapeutic regimens, such as rituximab and mycophenolate mofetil. Therefore, along with developing a novel therapeutic regimen, there is a need to identify a clinically useful biomarker for predicting the risk of all‐cause mortality in AAV patients during follow‐ups.

Body mass index (BMI) is a representative indicator of nutritional status and can be used to classify individuals into four categories: underweight, healthy weight, overweight, and obese. In the general population, overweight and obesity enhance the incidence rate of CVD as a component of metabolic syndrome and, therefore, are risk factors for all‐cause mortality.^6^, ^7^, ^8^, ^9^ Meanwhile, in critically ill patients, underweight is known to be an important risk factor for all‐cause mortality as malnutrition has negative effects on normal body functions, including vital organ fueling, tissue oxygenation, water/electrolyte balance, body temperature maintenance, and clearance of cellular debris.^10^, ^11^, ^12^

The conventional BMI is a two‐dimensional variable, which is higher than expected in taller individuals, and lower in those with shorter height.^13^ Recently, a new BMI has been proposed to overcome this limitation. It is a three‐dimensional variable and is calculated as follows: new BMI = 1.3 × weight (kg)/height (m) 2.5.^14^ Thus, it could be theoretically assumed that the new BMI at diagnosis can predict poor outcomes in patients with AAV during follow‐up. To the best of our knowledge, there are three studies that have applied the new BMI in clinical practice, with conflicting results.^15^, ^16^, ^17^ However, all of the studies were studies that analyzed postoperative outcomes, and there were no studies on AAV.

Hence, this study aimed to investigate the associations between the new BMI at diagnosis and poor outcomes during follow‐ups in patients with AAV. Additionally, this study compared the predictive power of conventional BMI for poor outcomes with that of the new BMI in patients with AAV.

## 2 METHODS

### 2.1 Patients

We retrospectively reviewed the medical records of 242 immunosuppressive drug‐naive patients with AAV. The patients were newly diagnosed with AAV based on the 2007 European Medicine Agency algorithms (the 2007 EMA algorithms) or the 2012 Chapel Hill Consensus Conference Nomenclature of Vasculitides (the 2012 CHCC definitions) between October 2000 and December 2020 at Severance Hospital, Yonsei University Health System, a tertiary medical center in Seoul, Republic of Korea.^2^, ^18^ According to the 2012 CHCC definitions, AAV is defined as necrotizing vasculitis with few or no immune deposits affecting small vessels associated with ANCAs. Specifically, GPA is defined as necrotizing granulomatous inflammation, MPA as necrotizing vasculitis without granulomatous inflammation, and EGPA as eosinophil‐rich necrotizing granulomatous inflammation associated with asthma and eosinophilia. The 2007 EMA algorithm is a flowchart of GPA, MPA, and EGPA for classifying systemic vasculitis. According to this algorithm, EGPA is to be classified first, and after EGPA is excluded, if there is a granuloma or GPA surrogate marker such as nasal or cartilage involvement, GPA is classified next. After excluding EGPA and GPA, MPA is classified according to clinical features, histology, and serological findings. The inclusion criteria of this study were as follows: (i) medical records well documented enough to assess AAV‐specific indices, namely the Birmingham Vasculitis Activity Score (BVAS) (version 3), an index used to assess disease activity,^19^ Five‐Factor Score (FFS), an index to evaluate the prognosis,^20^ and ANCA results and ii) the follow‐up duration of at least >3 months. The exclusion criteria were as follows: (i) serious medical conditions, such as malignancy, serious infection, and other systemic vasculitides, other than AAV and (ii) administration of immunosuppressive drugs for AAV treatment before diagnosis. This study was approved by the institutional review board of Severance Hospital (IRB protocol number 4‐2020‐1071). The need for written informed consent was waived owing to the retrospective nature of the study.

### 2.2 Definition of the new BMI

The new BMI was calculated using the following equation: new BMI = 1.3 × weight (kg)/height (m)^2.5^. All patients had their height (cm) and weight (kg) measured at diagnosis. According to the World Health Organization guidelines, the new BMI was categorized into four weight groups: underweight (<18.5 kg/m^2.5^), healthy weight (18.5 to <25.0 kg/m^2.5^), overweight (25.0 to <30.0 kg/m^2.5^), and obese (≥30.0 kg/m^2.5^).^21^

### 2.3 Clinical and laboratory data at diagnosis

We obtained baseline clinical and laboratory data, including complete blood count and biochemical tests, such as serum albumin, proteinuria, hematuria, myeloperoxidase (MPO)‐ANCA (or perinuclear [P]‐ANCA), and proteinase 3 (PR3)‐ANCA (or cytoplasmic [C]‐ANCA). The baseline characteristics and data on organ involvement; BVAS; FFS; and comorbidities, such as diabetes mellitus, hypertension, chronic kidney disease, and hyperlipidemia, were also collected.

### 2.4 Poor outcomes

We defined all‐cause mortality, relapse, end‐stage renal disease (ESRD) development, cerebrovascular accident (CVA), and CVD as poor outcomes of AAV. Relapse was defined as an active status of AAV after achieving remission. ESRD was defined as a medical condition requiring renal replacement therapy for >3 months. The follow‐up duration based on each poor outcome was defined as a period from the diagnosis to the time of the occurrence of each poor outcome. The follow‐up duration for patients without poor outcomes was defined as a period from diagnosis to the last follow‐up.

### 2.5 Statistical analyses

All statistical analyses were performed using SPSS software (version 25 for Windows; IBM Corp.). Continuous variables were expressed as median (interquartile range), and categorical variables were expressed as numbers and percentages. The Kruskal–Wallis test, chi‐squared test, Fisher's exact test, and Wilcoxon signed‐rank test were performed to evaluate the differences between the groups. The Kaplan–Meier curves and log‐rank test were used to investigate the association between the new BMI and outcomes. The correlation coefficient was obtained using Pearson correlation analysis. The Cox proportional hazards regression model was used to evaluate the hazard ratios (HRs) and 95% confidence intervals (CI) for poor outcomes in each weight group, with the healthy weight group as the reference. The multivariate Cox proportional hazards model analysis was performed using variables that were statistically significant in the univariate analysis. *p* Values of <0.05 were considered statistically significant.

## 3 RESULTS

### 3.1 Characteristics of patients with AAV

The baseline characteristics of patients and poor outcomes during the follow‐up are described in Table 1. The median age of the study population was 60.0 years, and 85 (35.1%) patients were males. MPA (54.1%) was the most common subtype of AAV, and 193 (79.8%) patients had ANCA. The median BVAS and FFS were 12.0 and 1.0, respectively. The median conventional BMI and new BMI were 22.6 kg/m^2^ and 23.3 kg/m^2.5^, respectively. During the follow‐up period of 35.9 months, 29 (12.0%) patients died of any cause, 80 (33.1%) experienced relapse, and 42 (17.4%) developed ESRD.

**TABLE 1:** Characteristics of the AAV patients at the time of diagnosis and during the follow‐up period (N = 242)

AAV patients	Values
At the time of diagnosis	
Demographic data	
Age (years)	60.0 (20.0)
Male sex (n (%))	85 (35.1)
AAV subtypes (n (%))	
MPA	131 (54.1)
GPA	62 (25.6)
EGPA	49 (20.2)
ANCA positivity (n (%))	
MPO‐ANCA (or P‐ANCA) positivity	163 (67.4)
PR3‐ANCA (or C‐ANCA) positivity	40 (16.5)
Both ANCA positivity	10 (4.1)
ANCA negativity	49 (20.2)
AAV‐specific indices	
BVAS	12.0 (11.0)
FFS	1.0 (1.0)
Clinical manifestations at diagnosis (n (%))	
General	102 (42.1)
Cutaneous	51 (21.1)
Muco‐membranous /Ocular	14 (5.8)
Ear–nose–throat	110 (45.5)
Pulmonary	110 (45.5)
Cardiovascular	54 (22.3)
Gastrointestinal	12 (5.0)
Renal	148 (61.2)
Nervous	81 (33.5)
Comorbidities at diagnosis (n (%))	
Chronic kidney disease (stage 3–5)	74 (30.6)
Diabetes mellitus	63 (26.0)
Hypertension	98 (40.5)
Hyperlipidemia	46 (19.0)
Interstitial lung disease	64 (26.4)
Routine laboratory results at diagnosis	
White blood cell count (/mm^3^)	9,180.0 (6,670.0)
Hemoglobin (g/dl)	11.3 (3.7)
Platelet count (×1000/mm^3^)	298.0 (161.0)
Fasting glucose (mg/dl)	101.0 (35.0)
BUN (mg/dl)	17.7 (21.7)
Serum creatinine (mg/dl)	0.9 (1.2)
Total protein (g/dl)	6.7 (1.2)
Serum albumin (g/dl)	3.6 (1.1)
ALP (IU/L)	71.0 (37.0)
AST (IU/L)	18.0 (9.0)
ALT (IU/L)	16.0 (14.0)
Total bilirubin (mg/dl)	0.5 (0.2)
ESR (mm/h)	59.0 (73.0)
CRP (mg/L)	13.8 (70.6)
Body mass index (kg/m^2^)	22.6 (4.4)
New body mass index (kg/m^2.5^)	23.3 (4.7)
During the follow‐up duration	
Follow‐up duration (months)	35.9 (235.9)
Poor outcomes during the follow‐up duration (n (%))	
All‐cause mortality (n (%))	29 (12.0)
Follow‐up duration based on all‐cause mortality (months)	35.9 (67.4)
Relapse (n (%))	80 (33.1)
Follow‐up duration based on relapse (months)	22.1 (42.8)
ESRD (n (%))	42 (17.4)
Follow‐up duration based on ESRD (months)	30.0 (64.6)
CVA (n (%))	18 (7.4)
Follow‐up duration based on CVA (months)	32.4 (63.7)
CVD (n (%))	11 (4.5)
Follow‐up duration based on CVD (months)	34.4 (64.2)
Medications administered during the follow‐up duration (n (%))	
Glucocorticoid	227 (93.8)
Cyclophosphamide	128 (52.9)
Rituximab	40 (16.5)
Azathioprine	130 (53.7)
Mycophenolate mofetil	29 (12.0)
Tacrolimus	16 (6.6)
Methotrexate	23 (9.5)

Note

Values are expressed as median (IQR) or number (percentage).

Abbreviations: AAV, ANCA‐associated vasculitis; ALP, alkaline phosphatase; ALT, alanine aminotransferase; ANCA, antineutrophil cytoplasmic antibody; AST, aspartate aminotransferase; BUN, blood urea nitrogen; BVAS, Birmingham vasculitis activity score; C, cytoplasmic; CRP, C‐reactive protein; CVA, cerebrovascular accident; CVD, cardiovascular disease; EGPA, eosinophilic GPA; ESR, erythrocyte sedimentation rate; ESRD, end‐stage renal disease; FFS, five‐factor score; GPA, granulomatosis with polyangiitis; IQR, interquartile range; MPA, microscopic polyangiitis; MPO, myeloperoxidase; P, perinuclear; PR3, proteinase 3.

### 3.2 Comparison of clinical characteristics among weight groups categorized according to the new BMI

We compared variables described in Table 1 among the four groups of patients who were categorized according to the new BMI. There were no differences in demographic and AAV‐specific data at diagnosis between the groups, except that the pulmonary involvement was more frequent in the underweight group than in other groups (*p* = 0.049). Diabetes and hyperlipidemia were more frequent in the obese group than in the other groups, as expected (*p* = 0.020 and 0.009, respectively). A higher proportion of patients in the underweight group developed ESRD (*p* = 0.004). No other significant differences were observed in the poor outcomes at follow‐ups between the groups (Table 2).

**TABLE 2:** Characteristics of patients with AAV according to the new BMI category

Variables	Underweight (<18.5 kg/m^2.5^) N = 16	Healthy weight (18.5 to <25.0 kg/m^2.5^) N = 151	Overweight (25.0 to <30.0 kg/m^2.5^) N = 65	Obese (≥30 kg/m^2.5^) N = 10	*p*‐Value
At the time of diagnosis					
Demographic data					
Age (years)	56.0 (36.3)	58.0 (22.0)	62.0 (13.0)	61.5 (29.5)	0.541
Conventional body mass index (kg/m^2^)	16.4 (1.1)	22.0 (3.1)	25.8 (2.1)	29.8 (2.1)	<0.001
Male sex (N, (%))	4 (25.0)	58 (38.4)	22 (33.8)	1 (10.0)	0.235
AAV subtypes (N, (%))					
MPA	1168.8)	81 (53.6)	36 (55.4)	3 (30.0)	0.506
GPA	2 (12.5)	39 (25.8)	18 (27.7)	3 (30.0)	
EGPA	3 (18.8)	31 (20.5)	11 (16.9)	4 (40.0)	
ANCA positivity (N, (%))					
MPO‐ANCA (or P‐ANCA) positivity	12 (75.0)	101 (66.9)	43 (66.2)	7 (70.0)	0.916
PR3‐ANCA (or C‐ANCA) positivity	1 (6.3)	26 (17.2)	9 (13.8)	4 (40.0)	0.132
Both ANCA positivity	0 (0.0)	7 (4.6)	2 (3.1)	10 (4.1)	0.607
ANCA negativity	3 (18.8)	31 (20.5)	15 (23.1)	0 (0.0)	0.409
AAV‐specific indices					
BVAS	16.0 (11.0)	12.0 (10.0)	12.0 (12.0)	17.0 (13.0)	0.101
FFS	2.0 (1.8)	1.0 (1.0)	1.0 (2.0)	1.0 (2.0)	0.447
Clinical manifestations at diagnosis (N, (%))					
General	9 (6.6)	151 (622.4)	65 (26.9)	10 (4.1)	0.415
Cutaneous	1 (6.3)	30 (19.9)	17 (26.2)	3 (30.0)	0.292
Muco‐membranous /Ocular	1(6.3)	9 (6.0)	3 (4.6)	1(10.0)	0.918
Ear nose throat	5 (31.3)	69 (45.7)	29 (44.6)	7 (70.0)	0.289
Pulmonary	13 (81.3)	86 (57.0)	42 (64.6)	3 (30.0)	**0.049**
Cardiovascular	5 (31.3)	29 (19.2)	19 (29.2)	1 (10.0)	0.236
Gastrointestinal	3 (18.8)	6 (4.0)	3 (4.6)	0 (0.0)	0.063
Renal	11 (68.8)	88 (58.3)	42 (64.6)	7 (70.0)	0.666
Nervous	4 (25.0)	53 (35.1)	19 (29.2)	5 (50.0)	0.485
Comorbidities at diagnosis (N, (%))					
Smoking history	0 (0.0)	6 (4.0)	3 (4.6)	0 (0.0)	0.758
Diabetes mellitus	2 (12.5)	34 (22.5)	21 (32.3)	6 (60.0)	**0.020**
Hypertension	8 (50.0)	60 (39.7)	26 (40.0)	4 (40.0)	0.886
Chronic kidney disease (stage 3–5)	5 (31.3)	50 (33.1)	16 (21.6)	3 (30.0)	0.671
Hyperlipidemia	2 (12.5)	26 (17.2)	12 (18.5)	6 (60.0)	**0.009**
Interstitial lung disease	3 (18.8)	37 (24.5)	22 (33.8)	2 (20.0)	0.420
Routine laboratory results at diagnosis					
White blood cell count (/mm^3^)	8695.0 (7155.0)	8910.0 (6805.0)	9360.0 (5540.0)	10,520.0 (6455.0)	0.748
Hemoglobin (g/dl)	10.2 (4.2)	11.4 (3.7)	11.3 (4.0)	12.4 (3.5)	0.660
Platelet count (×1000/mm^3^)	232.5 (166.0)	293.0 (148.0)	312.0 (178.0)	309.0 (205.5)	0.106
Fasting glucose (mg/dl)	106.5 (43.5)	101.0 (31.0)	102.0 (45.3)	109.0 (27.8)	0.808
BUN (mg/dl)	23.8 (42.7)	17.5 (19.0)	17.0 (17.7)	18.9 (17.4)	0.287
Serum creatinine (mg/dl)	1.1 (4.3)	0.9 (1.7)	1.0 (0.9)	1.0 (1.4)	0.296
Total cholesterol (mg/dl)	156.5 (96.5)	165.0 (62.8)	177.0 (68.0)	175.0 (49.0)	0.566
Total protein (g/dl)	6.9 (1.6)	6.8 (1.2)	6.5 (1.1)	6.6 (0.7)	0.652
Serum albumin (g/dl)	3.7 (1.4)	3.7 (1.0)	3.5 (1.3)	3.4 (1.4)	0.935
AST (IU/L)	18.0 (6.5)	18.0 (9.0)	18.0 (9.5)	21.0 (12.5)	0.783
ALT (IU/L)	15.5 (12.3)	15.0 (13.3)	19.0 (16.0)	14.0 (18.5)	0.372
Total bilirubin (mg/dl)	0.5 (0.1)	0.5 (0.3)	0.4 (0.3)	0.5 (0.2)	0.182
ESR (mm/h)	68.5 (63.8)	59.0 (68.3)	69.0 (86.5)	41.0 (64.5)	0.312
CRP (mg/L)	11.5 (114.0)	12.3 (69.8)	22.0 (70.3)	4.3 (55.3)	0.418
During the follow‐up period					
Follow‐up duration (months)	58.2 (55.1)	31.8 (72.8)	40.0 (59.3)	67.2 (54.1)	0.418
Poor outcomes during follow‐up (N, (%))					
All‐cause mortality (N, (%))	5 (31.3)	16 (10.6)	8 (12.3)	0 (0.0)	0.064
Follow‐up duration based on all‐cause mortality (months)	57.4 (54.1)	32.4 (77.3)	40.0 (57.3)	66.7 (53.8)	0.518
Relapse (N, (%))	9 (56.3)	50 (33.1)	18 (27.7)	3 (30.0)	0.189
Follow‐up duration based on relapse (months)	16.4 (45.9)	21.8 (41.8)	22.1 (45.3)	40.3 (51.9)	0.299
ESRD (N, (%))	7 (43.8)	29 (19.2)	6 (9.2)	0 (0.0)	**0.004**
Follow‐up duration based on ESRD (months)	16.5 (57.8)	26.9 (73.6)	39.5 (59.4)	66.7 (53.8)	0.053
CVA (N, (%))	1 (6.3)	10 (6.6)	7 (10.8)	0 (0.0)	0.566
Follow‐up duration based on CVA (months)	44.5 (60.8)	29.1 (71.8)	36.2 (53.7)	67.2 (54.1)	0.328
CVD (N, (%))	1 (6.3)	4 (2.6)	5 (7.7)	1 (10.0)	0.317
Follow‐up duration based on CVD (months)	58.2 (50.3)	31.0 (70.6)	38.2 (59.4)	67.2 (46.7)	0.417
Medications administered during follow‐up (N, (%))					
Glucocorticoid	16 (100.0)	142 (94.0)	59 (90.8)	10 (100.0)	0.430
Cyclophosphamide	8 (50.0)	86 (57.0)	30 (46.2)	4 (40.0)	0.407
Rituximab	3 (18.8)	27 (17.9)	9 (13.8)	1 (10.0)	0.824
Azathioprine	4 (25.0)	83 (55.0)	34 (52.3)	9 (90.0)	**0.013**
Mycophenolate mofetil	5 (31.3)	20 (13.2)	3 (4.6)	1 (10.0)	**0.026**
Tacrolimus	3 (18.8)	10 (6.6)	3 (4.6)	0 (0.0)	0.176
Methotrexate	0 (0.0)	15 (9.9)	6 (9.2)	2 (20.0)	0.392

Note

Values are expressed as median (interquartile range, IQR) or N (%).

Abbreviations: AAV, ANCA‐associated vasculitis; ALT, alanine aminotransferase; ANCA, antineutrophil cytoplasmic antibody; AST, aspartate aminotransferase; BUN, blood urea nitrogen; BVAS, Birmingham vasculitis activity score; C, cytoplasmic; CRP, C‐reactive protein; EGPA, eosinophilic GPA; ESR, erythrocyte sedimentation rate; FFS, five‐factor score; GPA, granulomatosis with polyangiitis; mBMI, modified body mass index; MPA, microscopic polyangiitis; MPO, myeloperoxidase; P, perinuclear; PR3, proteinase 3.

### 3.3 Association between poor outcomes and weight groups categorized according to new and conventional BMI

The HRs for poor outcomes were calculated in the underweight, overweight, and obese groups, categorized according to the new and conventional BMIs (Table 3). The underweight group according to new BMI exhibited significantly increased risk for all‐cause mortality (HR 3.180, 95% CI 1.134–8.922), relapse (HR 2.141, 95% CI 1.019–4.368), and ESRD occurrence (HR 2.729, 95% CI 1.190–6.259) compared to the healthy weight group. However, the risks for poor outcomes were not statistically significant in the overweight and obesity groups than in the healthy weight group. Moreover, adjusted HRs were analyzed by adjusting the variables which showed significant differences for each outcomes. As a result, underweight group according to the new BMI showed significantly increased risk for all‐cause mortality and relapse than healthy weight group, however, the risk for ESRD occurrence lost the significance.

**TABLE 3:** Hazard ratios (HR) for poor outcomes according to the weight categories by new body mass index (BMI) and conventional BMI

	New BMI				Conventional BMI			
	Underweight (<18.5 kg/m^2.5^) N = 16	Healthy weight (18.5 to <25.0 kg/m^2.5^) N = 151	Overweight (25.0 to <30.0 kg/m^2.5^) N = 65	Obese (≥30 kg/m^2.5^) N = 10	Underweight (<18.5 kg/m^2.5^) N = 24	Healthy weight (18.5 to <25.0 kg/m^2.5^) N = 165	Overweight (25.0 to <30.0 kg/m^2.5^) N = 48	Obese (≥30 kg/m^2.5^) N = 5
All‐cause mortality HR	3.180	Ref	1.262	0.045	2.191	Ref	0.773	0.047
95% CI	1.134–8.922		0.537–2.963	<0.001–224.952	0.853–5.630		0.302–1.976	<0.001–2847.816
*p*‐Value	**0.028**		0.593	0.475	0.103		0.591	0.585
Adjusted all‐cause mortality HR	3.649	Ref	0.817	<0.001	2.797	Ref	2.190	<0.001
95% CI	1.132–11.764		0.288–2.314	<0.001	0.970–8.068		0.786–6.098	<0.001
*p*‐Value	**0.030**		0.703	0.984	0.057		0.134	0.986
Relapse HR (95% CI)	2.141	Ref	0.786	0.726	1.707	Ref	0.746	0.975
95% CI	1.019–4.368		0.459–1.348	0.226–2.330	0.928–3.141		0.406–1.371	0.237–4.008
*p*‐Value	**0.036**		0.382	0.590	0.086		0.345	0.971
Adjusted relapse HR (95% CI)	2.453	Ref	0.774	0.620	2.016	Ref	0.764	0.668
95% CI	1.185–5.079		0.451–1.327	0.192–2.003	1.245–4.004		0.416–1.406	0.158–2.828
*p*‐Value	**0.016**		0.352	0.424	**0.029**		0.387	0.584
ESRD HR (95% CI)	2.729	Ref	0.481	0.044	1.698	Ref	0.196	0.046
95% CI	1.190–6.259		0.179–1.289	<0.001–18.676	0.781–3.691		0.047–0.820	<0.001–110.103
*p*‐Value	**0.018**		0.146	0.311	0.182		**0.026**	0.438
Adjusted ESRD HR (95% CI)	1.543	Ref	0.526	<0.001	1.320	Ref	0.404	<0.001
95% CI	0.575–4.145		0.194–1.429	<0.001	0.573–3.038		0.093–1.761	<0.001
*p*‐Value	0.389		0.208	0.976	0.514		0.228	0.977
CVA HR (95% CI)	0.043	Ref	1.570	0.044	0.040	Ref	0.779	0.047
95% CI	<0.001–678.758		0.558–4.402	<0.001–3347.009	<0.001–53.205		0.220–2.765	<0.001–59,116.900
*p*‐Value	0.523		0.394	0.584	0.380		0.699	0.670
Adjusted CVA HR (95% CI)	<0.001	Ref	1.261	<0.001	<0.001	Ref	0.952	<0.001
95% CI	<0.001		0.425–3.745	<0.001	<0.001		0.262–3.461	<0.001
*p*‐Value	0.985		0.676	0.988	0.983		0.941	0.986
CVD HR	3.02	Ref	4.007	4.007	1.177	Ref	3.415	5.299
95% CI	0.301–30.188		0.953–16.847	0.441–39.033	0.130–10.666		0.847–13.764	0.579–48.462
*p*‐Value	0.347		0.058	0.232	0.885		0.084	0.140
Adjusted CVD HR	2.916	Ref	3.335	3.819	1.078	Ref	4.397	4.736
95% CI	0.283–30.007		0.778–14.304	0.390–37.378	0.119–9.806		1.053–18.361	0.499–44.919
*p*‐Value	0.368		0.105	0.250	0.947		**0.042**	0.175

Abbreviations: CI, confidence interval; CVA, cerebrovascular accident; CVD, cardiovascular disease; ESRD, end‐stage renal disease.

On the contrary, in the weight groups categorized according to conventional BMI, the ESRD occurrence was significantly associated with the overweight group (HR 0.196, 95% CI 0.047–0.820). No other significant associations between poor outcomes and the three groups (underweight, overweight, and obesity) were observed.

### 3.4 Incidence rates of poor outcomes

**FIGURE 1:**
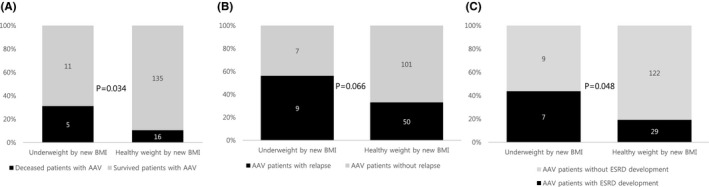
Comparison of the incidence rates of poor outcomes between the underweight and healthy weight groups by the new body mass index (BMI). The underweight group exhibited higher incidence rates of both all‐cause mortality (31.3% vs. 10.6%, *p* = 0.034) and ESRD occurrence (43.8% vs. 19.2%, *p* = 0.048) than the healthy weight group. The incidence rate of relapse did not differ between the two groups

Since significant associations were found between the underweight group and poor outcomes, we divided the patients into the underweight and healthy weight groups according to the ranges of the new BMI. The underweight group exhibited higher incidence rates than the healthy weight group for both all‐cause mortality (31.3% vs. 10.6%, *p* = 0.034) and ESRD occurrence (43.8% vs. 19.2%, *p* = 0.048). However, the incidence rate for relapse did not differ between the two groups (Figure 1). Additionally, there were no differences in the incidence rates of CVA and CVD between the two groups.

### 3.5 Kaplan–Meier analysis for poor outcomes in underweight patients with AAV according to the new BMI

**FIGURE 2:**
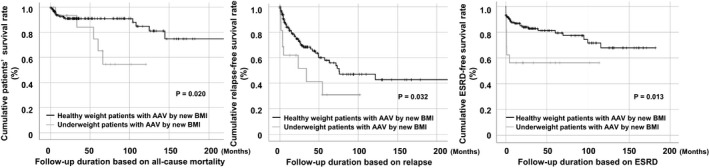
Kaplan–Meier survival curve for poor outcomes stratified underweight and healthy weight by the new body mass index (BMI). Patients assigned to the underweight group based on the new BMI exhibited lower cumulative patients' (*p* = 0.020), relapse‐free (*p* = 0.032), and ESRD‐free survival rates (*p* = 0.013) than those assigned to the healthy weight group based on the new BMI

Based on the new BMI, patients in the underweight group exhibited lower cumulative (*p* = 0.020), relapse‐free (*p* = 0.032), and ESRD‐free survival rates (*p* = 0.013) than those in the healthy weight group (Figure 2).

### 3.6 Cox proportional hazards model analyses

We performed the Cox proportional regression analysis to investigate whether the underweight category defined according to the new BMI could independently predict all‐cause mortality. In the univariate analysis, age, male sex, BVAS, FFS, interstitial lung disease, serum creatinine levels, serum albumin levels, C‐reactive protein (CRP) levels, and underweight based on the new BMI category at diagnosis were associated with all‐cause mortality during the follow‐up. In the multivariate analysis, underweight based on new BMI (HR 5.285), age (HR 1.057), male sex (HR 3.595), BVAS (HR 1.161), FFS (HR 2.957), interstitial lung disease (HR 31.874), and serum albumin levels (HR 0.134) were independent predictors of all‐cause mortality. However, the underweight category defined by the conventional BMI at diagnosis was not significantly associated with all‐cause mortality during the follow‐up (Table 4). Underweight based on the new BMI was associated with relapse in the univariate (HR 2.4553) and multivariate analyses (HR 2.453) (Table S1). It was also a risk factor for ESRD development (HR 2.729) in univariate analysis; however, in the multivariate analysis, after adjusting for variables and including factors associated with the ESRD development in AAV, no significant association was found (Table S2). On the other hand, patients defined as underweight according to the conventional BMI at diagnosis were not associated with relapse or ESRD development.

**TABLE 4:** Cox regression analysis of the variables associated with all‐cause mortality

Variables	Univariable			Multivariable		
	HR	95% CI	*p*‐Value	HR	95% CI	*p*‐Value
Age (years)	1.056	1.022–1.091	**0.001**	1.057	1.015–1.101	**0.007**
Male sex (n, (%))	2.181	1.049–4.535	**0.037**	3.595	1.245–10.384	**0.018**
MPO‐ANCA (or P‐ANCA) positivity	1.435	0.646–3.187	0.375			
PR3‐ANCA (or C‐ANCA) positivity	0.811	0.308–2.138	0.672			
BVAS	1.096	1.043–1.151	**<0.001**	1.161	1.070–1.260	**<0.001**
FFS	2.169	1.531–3.074	**<0.001**	2.957	1.506–5.807	**0.002**
Chronic kidney disease (stage 3–5)	1.865	0.896–3.881	0.096			
Diabetes mellitus	1.061	0.469–2.400	0.886			
Hypertension	0.923	0.440–1.936	0.833			
Hyperlipidemia	1.463	0.625–3.428	0.381			
Interstitial lung disease	2.674	1.283–5.574	**0.009**	31.874	8.591–118.260	**<0.001**
Serum creatinine (mg/dl)	1.152	1.008–1.317	**0.037**	0.878	0.689–1.120	0.296
Serum albumin (g/dl)	0.356	0.213–0.596	**<0.001**	0.134	0.039–0.459	**0.001**
ESR (mm/h)	1.006	0.997–1.015	0.193			
CRP (mg/L)	1.007	1.002–1.012	**0.008**	0.996	0.996–1.007	0.458
New BMI <18.5 kg/m^2^	3.180	1.134–8.922	**0.028**	5.285	1.468–19.018	**0.011**
Conventional BMI <18.5 kg/m^2^	2.191	0.853–5.630	0.103			

Note

Values are expressed as mean ± standard deviation or number (percentage).

Abbreviations: AAV, ANCA‐associated vasculitis; ANCA, antineutrophil cytoplasmic antibody; BMI, body mass index; BVAS, Birmingham vasculitis activity score; C, cytoplasmic; CI, confidence interval; CRP, C‐reactive protein; ESR, erythrocyte sedimentation rate; FFS, five‐factor score; HR, hazard ratio; MPO, myeloperoxidase; P, perinuclear; PR3, proteinase 3.

Bold indicates statistically significant.

## 4 DISCUSSION

Our study demonstrated that patients categorized in the underweight group based on the new BMI at the time of AAV diagnosis had significantly higher incidence of all‐cause mortality, relapse, and ESRD development as compared to those in the healthy weight group.

Furthermore, our data demonstrated that underweight based on the new BMI may be a significant and independent predictor for all‐cause mortality in patients with AAV during follow‐ups. To the best of our knowledge, ours is the first study to identify the association between the new BMI and poor outcomes in AAV.

Previous studies have demonstrated that in critical conditions, underweight patients were at a higher risk of mortality.^22^, ^23^, ^24^, ^25^ Since AAV is a fatal disease that may result in critical illness if not properly treated, we hypothesized that underweight is associated with poor outcomes in AAV. Some may argue with this hypothesis, asserting that underweight is not a prognostic factor, rather is an epiphenomenon of the disease. However, our results indicated no correlation between the new BMI at diagnosis and baseline BVAS, FFS, or CRP levels. Furthermore, adjusted HRs demonstrated that the new BMI was associated with mortality independently of age, baseline BVAS, FFS, and CRP levels. Therefore, it is reasonable to suggest that underweight, an indicator of nutritional status, is a poor prognostic factor in patients with AAV.

Malnutrition significantly increases mortality in critically ill patients^24^ and in those with chronic diseases.^26^, ^27^ In critical illness, energy usage is prioritized for vital organs, such as the brain or heart, and nutrients stored in the muscle or adipose tissue are catabolized to produce energy substrates.^28^ Furthermore, inflammatory cytokines, such as interleukin (IL)‐1, IL‐6, and tumor necrosis factor‐α, accelerate the catabolic process.^29^ However, underweight patients may not have enough nutrient reserves; therefore, they are susceptible to malnutrition. Based on these findings, it is reasonable to assume that underweight patients with AAV would have worse outcomes than healthy weight patients; however, there were no previous data regarding the association between underweight and AAV. Assuming that the new BMI reflects the actual shape of the human body, our finding is significant in revealing the association between underweight and poor outcomes in patients with AAV.

Underweight patients, according to the new BMI at diagnosis, experienced more frequent relapse and ESRD development than healthy weight patients. The exact mechanism underlying these associations is unclear. Our team had previously evaluated prognostic nutritional index, an index that reflects nutritional status, in patients with AAV and revealed that it correlated with the BVAS and predicted relapse.^30^ Similarly, we demonstrated that a poor nutritional status was associated with a higher BVAS, and that it was a risk factor for ESRD occurrence.^31^ One explanation for these findings is that underweight patients are at an increased risk of low muscle mass,^32^, ^33^ and muscle mass is associated with chronic kidney disease.^34^, ^35^

The new BMI was proposed since the conventional BMI, which is defined as weight divided by height squared, has limitations in reflecting the three‐dimensional human body. The weight is divided too much for short people and too little for tall people, resulting in lower BMI for short people and higher BMI for tall people. Therefore, the new BMI formula by Professor Trefethen could provide a closer approximation to real human shape. Previous studies showed that male patients shifted to lower BMI category by new BMI compared to conventional BMI, which can be explained by the effect of height.^15^, ^17^ We can interpret that the new BMI improved the limit of the conventional BMI, which is calculated to be more obese in tall people.

In the present study, underweight, according to the new BMI at diagnosis, was a poor prognostic factor in patients with AAV; however, this result could not be achieved using conventional BMI. Since there is increasing evidence demonstrating underweight as a risk factor for mortality in critically ill patients, as previously described, our finding may suggest that the new BMI is superior to the conventional BMI in reflecting the actual human body shape.

Our study has some limitations. First, we could not analyze the precise precipitating events in patients with poor outcomes, which makes it difficult to determine the reason why underweight patients showed more poor outcomes. There were limitations in determining whether underweight patients were more vulnerable to toxic immunosuppressants, more susceptible to infections, or negatively affected by AAV itself. Second, owing to the retrospective study design, we could not obtain more information on muscle and fat masses. Third, all patients in our study were Korean, which makes it difficult to generalize our findings. Further studies are required to assess and validate our findings in other AAV cohorts with various ethnicities.

Our study has several strengths. First, this is the first study to report the association of underweight according to the new BMI with the poor outcomes in patients with AAV. Second, our study confirmed the difference between the new BMI and conventional BMI, suggesting that the new BMI may be superior to the conventional BMI in reflecting real human body shape. Finally, our study revealed a simple but important finding that underweight, according to the new BMI, may be used as an independent prognostic factor in patients with AAV.

In conclusion, our study demonstrated the association between underweight, according to the new BMI, and poor outcomes in patients with AAV. Based on our results, we suggest evaluating the nutritional status of patients with AAV during diagnosis using the new BMI as a prognostic factor. Further prospective studies are required to validate our findings and to establish the underlying mechanisms of our observed findings.

## CONFLICT OF INTEREST

The authors declare that they have no conflicts of interests.

## AUTHOR CONTRIBUTIONS

JYP, SSJ, YBP, and SWL participated in research design, the writing of the final version of manuscript, and the performance of the research. JYP, SSA, LEL, and SWL contributed to the acquisition of data and interpretations of data. JYP, SSA, and SWL participated in the preparation of the draft manuscript. HNC conducted the statistical analyses and validated the interpretation. In particular, LEL helped with English editing. All authors read and approved the final revision of the manuscript.

## Supporting information

Table S1

Table S2

## Data Availability

The datasets of the current study are available from the corresponding author on reasonable request.
