# Supplementary material for: New body mass index for predicting prognosis in patients with antineutrophil cytoplasmic antibody‐associated vasculitis
Source: J Clin Lab Anal. 2022 Mar 21;36(5):e24357. doi: 10.1002/jcla.24357 (PMC9102757; doi:10.1002/jcla.24357)
Supplement: Supplementary file 1 — Table S1 [file JCLA-36-e24357-s001.docx]

**Supplementary Table S1. Cox regression analysis of the variables associated with relapse**

| **Variables** | **Univariable** | | |  | **Multivariable** | | |
| --- | --- | --- | --- | --- | --- | --- | --- |
|  | **HR** | **95% CI** | **P-value** |  | **HR** | **95% CI** | **P-value** |
| Age (years) | 0.987 | 0.972-1.001 | 0.065 |  |  |  |  |
| Male sex (n, (%)) | 1.378 | 0.872-2.179 | 0.170 |  |  |  |  |
| MPO-ANCA (or P-ANCA) positivity | 1.163 | 0.729-1.855 | 0.525 |  |  |  |  |
| PR3-ANCA (or C-ANCA) positivity | 2.217 | 1.366-3.603 | 0.001 |  | 2.136 | 1.157-3.942 | 0.015 |
| BVAS | 1.026 | 0.992-1.061 | 0.140 |  |  |  |  |
| FFS | 1.094 | 0.883-1.356 | 0.411 |  |  |  |  |
| Chronic kidney disease (stage 3–5) | 1.266 | 0.799-2.005 | 0.314 |  |  |  |  |
| Diabetes mellitus | 0.792 | 0.469-1.340 | 0.385 |  |  |  |  |
| Hypertension | 1.358 | 0.876-2.107 | 0.172 |  |  |  |  |
| Hyperlipidemia | 0.930 | 0.530-1.632 | 0.802 |  |  |  |  |
| Interstitial lung disease | 1.299 | 0.800-2.110 | 0.291 |  |  |  |  |
| Serum creatinine (mg/dL) | 1.063 | 0.960-1.177 | 0.239 |  |  |  |  |
| Serum albumin (g/dL) | 0.893 | 0.72-1.187 | 0.438 |  |  |  |  |
| ESR (mm/h) | 1.002 | 0.997-1.008 | 0.408 |  |  |  |  |
| CRP (mg/L) | 1.1001 | 0.997-1.005 | 0.599 |  |  |  |  |
| New BMI <18.5 kg/m^2.5^ | 2.141 | 1.049-4.368 | 0.036 |  | 2.453 | 1.185-5.079 | 0.016 |
| Conventional BMI <18.5 kg/m^2^ | 1.707 | 0.928-3.141 | 0.086 |  |  |  |  |

Values are expressed as means±standard deviations or numbers (percentages).

HR, hazard ratio; CI, confidence interval.

AAV: ANCA-associated vasculitis; ANCA: antineutrophil cytoplasmic antibody; MPO: myeloperoxidase; P: perinuclear; PR3: proteinase 3; C: cytoplasmic; BVAS: Birmingham vasculitis activity score; FFS: five factor score; ESR: erythrocyte sedimentation rate; CRP: C-reactive protein; BMI: body mass index.
