# Supplementary material for: New body mass index for predicting prognosis in patients with antineutrophil cytoplasmic antibody‐associated vasculitis
Source: J Clin Lab Anal. 2022 Mar 21;36(5):e24357. doi: 10.1002/jcla.24357 (PMC9102757; doi:10.1002/jcla.24357)
Supplement: Supplementary file 2 — Table S2 [file JCLA-36-e24357-s002.docx]

**Supplementary Table S2. Cox regression analysis of the variables associated with ESRD development**

| **Variables** | **Univariable** | | |  | **Multivariable** | | |
| --- | --- | --- | --- | --- | --- | --- | --- |
|  | **HR** | **95% CI** | **P-value** |  | **HR** | **95% CI** | **P-value** |
| Age (years) | 1.013 | 0.991-1.036 | 0.236 |  |  |  |  |
| Male sex (n, (%)) | 0.933 | 0.484-1.799 | 0.836 |  |  |  |  |
| MPO-ANCA (or P-ANCA) positivity | 2.474 | 1.141-5.365 | 0.022 |  | 1.014 | 0.439-2.347 | 0.973 |
| PR3-ANCA (or C-ANCA) positivity | 0.709 | 0.297-1.692 | 0.439 |  |  |  |  |
| BVAS | 1.079 | 1.033-1.125 | 0.001 |  | 1.027 | 0.972-1.085 | 0.343 |
| FFS | 1.993 | 1.492-2.663 | <0.001 |  | 1.456 | 1.013-2.092 | 0.042 |
| Chronic kidney disease (stage 3–5) | 0.991 | 0.515-1.907 | 0.978 |  |  |  |  |
| Diabetes mellitus | 0.901 | 0.443-1.834 | 0.774 |  |  |  |  |
| Hypertension | 1.980 | 1.074-3.651 | 0.029 |  | 1.138 | 0.563-2.300 | 0.720 |
| Hyperlipidemia | 1.193 | 0.571-2.494 | 0.639 |  |  |  |  |
| Interstitial lung disease | 0.686 | 0.317-1.484 | 0.338 |  |  |  |  |
| Serum creatinine (mg/dL) | 1.639 | 1.501-1.789 | <0.001 |  | 1.597 | 1.433-1.780 | <0.001 |
| Serum albumin (g/dL) | 0.576 | 0.386-0.861 | 0.007 |  | 0.704 | 0.409-1.212 | 0.205 |
| ESR (mm/h) | 1.007 | 0.999-1.014 | 0.077 |  |  |  |  |
| CRP (mg/L) | 1.004 | 0.999-1.008 | 0.139 |  |  |  |  |
| New BMI <18.5 kg/m^2.5^ | 2.729 | 1.190-6.259 | 0.018 |  | 1.320 | 0.573-3.038 | 0.514 |
| Conventional BMI <18.5 kg/m^2^ | 1.698 | 0.781-3.691 | 0.182 |  |  |  |  |

Values are expressed as means±standard deviations or numbers (percentages).

HR, hazard ratio; CI, confidence interval.

AAV: ANCA-associated vasculitis; ANCA: antineutrophil cytoplasmic antibody; MPO: myeloperoxidase; P: perinuclear; PR3: proteinase 3; C: cytoplasmic; BVAS: Birmingham vasculitis activity score; FFS: five factor score; ESR: erythrocyte sedimentation rate; CRP: C-reactive protein; BMI: body mass index.
